# Expression of ALDH and SOX-2 in Pulmonary Sclerosing Pnemocytoma (PSP) of the Lung: Is There a Meaning Behind?

**DOI:** 10.3389/fmed.2020.00497

**Published:** 2020-09-02

**Authors:** Beatrice Aramini, Valentina Masciale, Beatrice Manfredini, Daniel Bianchi, Federico Banchelli, Roberto D'Amico, Federica Bertolini, Massimo Dominici, Uliano Morandi, Antonino Maiorana

**Affiliations:** ^1^Division of Thoracic Surgery, Department of Medical and Surgical Sciences, University of Modena and Reggio Emilia, Modena, Italy; ^2^Department of Medical and Surgical Sciences, Center of Statistic, University of Modena and Reggio Emilia, Modena, Italy; ^3^Division of Oncology, Department of Medical and Surgical Sciences, University of Modena and Reggio Emilia, Modena, Italy; ^4^Department of Medical and Surgical Sciences, Institute of Pathology, University of Modena and Reggio Emilia, Modena, Italy

**Keywords:** pulmonary slerosing pneumocytoma, benign disease, lung rare disease, ALDH, SOX-2

## Abstract

**Background:** Pulmonary sclerosing pneumocytoma (PSP) is a rare benign pulmonary tumor that derives from primitive respiratory epithelium of the pulmonary alveolus. The etiology and pathogenesis are still unclear. Histopathological diagnosis focuses on cells that are positive for TTF1, EMA, cytokeratin-7, and CAM 5.2. The aim of our study is to highlight the elevated expression of ALDH and the presence of SOX-2 in pulmonary sclerosing pneumocytoma.

**Methods:** We report five cases of pulmonary sclerosing pneumocytoma undergone surgery at our Division of Thoracic Surgery, during a period between 1994 and 2011. ALDH and SOX-2 markers were also tested for positivity in all the patients.

**Results:** Patients showed elevated expression of ALDH during immunohistochemistry and mild expression of SOX-2, although in two cases in which SOX-2 was highly expressed. Among these two patients, one presented with lymph node recurrence while the other had no recurrence with a PET-positive nodule. In particular, the patient who had developed recurrence had an ALDH score of 4 and a SOX-2 score of 3, whereas the patient with the PET-positive nodule showed an ALDH score of 4 with a mild SOX-2 expression of score 1.

**Conclusions:** This is the first attempt demonstrating the elevated expression of ALDH in this disease. SOX-2 expression was noted in both the patient who developed recurrence and the patient with a PET-positive nodule. We believe that further investigation may be highly useful to better characterize these two markers as well as understand their function.

## Introduction

Pulmonary sclerosing pneumocytoma (PSP) is a rare benign tumor in the lung (1). This disease was firstly described by Liebow and Hubell1 as sclerosing hemangioma of the lung owing to prominent sclerotization and vascularization of the tissue. Pathologically, sclerosing hemangioma (SH) is typically composed of solid, papillary, sclerotic, or hemangiomatous components (2). The term “sclerosing hemangioma” was recently changed in 2015 by the WHO Classification of Lung Tumors as “pulmonary sclerosing pneumocytoma” (3). PSP was thought to be a vascular tumor but greater knowledge of this disease has led scientists to reconsider PSP as derived from primitive respiratory epithelium of the pulmonary alveolus, principally in type II alveolar cells (4). However, vascularization of this tumor is one of the main characteristics (4). PSP is difficult to recognize due to the lack of significant clinical or imaging features and at first glance appears to be a benign nodule such as hamartoma, tuberculoma, bronchial cysts, or certain lung cancer nodules (5). Although the etiology and pathogenesis of PSP are still unclear, recently it has been clarified that PSP is derived from the epithelial part of the lung, which gives rise to the name sclerosing pneumocytoma of the lung (6). PSPs are composed of four major histologic patterns, which are hemangiomatous, papillary, sclerotic, and solid, as well as different radiological characteristics made apparent by computed tomography (CT) findings according to their composition (6–14). It has been described that PSP typically presents as a well-defined, juxta-pleural nodule with strong and homogeneous enhancement (14–16) on CT, while several studies have analyzed CT findings of PSP and its pathologic correlation (14, 15). However, there is a limitation due to the small number of patients and the preoperative imaging modalities used in clinical practice to diagnosis PSP (16–18). In addition, a few studies investigated PSP using 18F-fluorodeoxyglucose positron emission tomography (FDG PET) (19, 20). The interval between the PET and CT study was a mean of 3.2 months (5–10); however, the hypermetabolism in pneumocytoma has not yet been defined or correlated with the possibility of developing into a malignant disease.

Patients are usually asymptomatic in which the PSP is detected coincidentally. Moreover, a cough, chest pain, and haemoptysis may also occur (6). PSP is often a solitary, well-defined, round or oval, homogeneous nodule, or mass (14–16); however, there are also cases of metastases to the lymph nodes, pleura, and bones (14, 17, 18). Patients may present with a mass lesion of up to 7 cm although 73% of lesions measure under 3 cm (8, 10). Marginal pseudocapsules (50%), overlying vessels (26.3%), air gap (2.6%), and halo sign (17.1%) are among thoracic CT findings (12). Occasionally, pleural-based, polypoid lesions may mimic a solitary fibrous tumor. Often the tumor consists of superficial cuboidal and round interstitial cells with a combination of four patterns. In the case of a biopsy with a predominance of papillary components of the sclerosing pneumocytoma, diagnosis may be difficult. In addition, both superficial cuboidal cells and round interstitial cells are positively immunoreactive for TTF1 and EMA (10, 19). TTF1 is used in the diagnosis of lung adenocarcinoma and may be misleading for diagnosing PSP. Napsin A, a human aspartic proteinase, shows immunohistochemical reactivity in type II pneumocytes with a granular and cytoplasmic staining pattern (18). It has been widely used in the panel for diagnosis of lung adenocarcinoma along with TTF-1 (17). Recently it has been demonstrated that Napsin A preferentially stains cuboidal surface cells, rather than stromal round cells, in sclerosing pneumocytoma (20, 21). Round cells are generally uniformly negative for pan-cytokeratin and positive for cytokeratin-7 and CAM 5.2 in few cases (10). However, markers related to possible malignancy transformation have not yet been identified.

The idea to test ALDH and SOX2 markers derives from the consideration of the pathological characteristics of this tumor constituted by an abundant vascularized component, and for the roles that ALDH and SOX2 may have in the process of endothelial vascular tumor progression. In particular, it has been shown that ALDH is able to detect endothelial stem-like cells in tumor with a role into the angiogenetic process (22), although SOX2 is involved into the endothelial differentiation process since this transcription factor have been shown to be essential mediators in vascular development (23, 24).

For these considerations, the aim of our study is to highlight the elevated expression of ALDH in the pneumocytoma of the lung as described, as well as the presence of SOX-2 markers in patients with a lymph node recurrence, and in one with a PET-positive nodule. However, we are conscious of the limitation of our study for the low number of patients. If the presence of these two markers will be confirmed in a larger cohort, it would be helpful for the setting of future target therapies, which may avoid the patient from lung resection for a benign disease.

## Materials and Methods

We describe five patients (all female with a mean age of 54 years) who underwent surgery in our department between 1994 and 2011 for a pulmonary sclerosing pneumocytoma of the lung. All patients were subjected to major lung resection. The diagnosis of pneumocytoma has been carried out by morphological evaluation and immunostaining of transcription termination factor 1 (TTF-1), pan-citocheratine (MNF116), and epithelial membrane antigen (EMA) (3). No postoperative complications were noted. Patients have been checked by the oncologist for the definition of signs or symptoms for the disease and for the follow-up. Data analysis was carried out by reporting descriptive statistics of the study sample.

### Immunohistochemistry and Microscopic Evaluation

Immunohistochemical stains were performed on formalin-fixed, paraffin-embedded 5 μm sections. The sections were first deparaffinized in xylene and further heated for 15 min in a 95°C water bath with a 10 mM sodium citrate buffer, then washed in PBS until the buffer had cooled. Incubation in 3% H_2_O_2_ at room temperature for 10 min was performed. Slides were then blocked with 10% goat serum in TBS for 15 min in order to reduce non-specific background. Sectional incubation with rabbit monoclonal mouse anti-ALDH (1:100) (ab−134188; Abcam, Cambridge, MA, USA) and mouse monoclonal anti-SOX-2 (1:200) (MA1-014 Thermo Fisher Scientific, Meridian Road Rockford, IL, USA) was performed overnight at 4°C. Specimens were washed in PBS and incubated with a biotinylated secondary antibody (PK-4001; Vector Labs, USA) for 30 min at room temperature and then stained with 3,3'-Diaminobenzidine tetrahydrochloride (DAB) after which tissue sections were counterstained with Mayer's Hematoxylin. A secondary antibody-only staining sample was used as a background control to evaluate positivity for ALDH and SOX-2, respectively. Images were collected and positivity was evaluated by a Zeiss AxioCam ICc 3 High-Resolution through an Axioskop microscope camera. For the assessment, section samples were investigated employing a semi-quantitative method using a score value for the positivity of the markers. Shown here are the score classes: 0 (<5% positive), 1 (5–25% positive), 2 (>25–50% positive), 3 (>50–75% positive), and 4 (>75% positive) (25). Sections were scored by two trained investigators who were blinded to the outcome of the patient and other clinical findings.

## Results

### Clinical Results

All patients were free from the disease after surgery and received a follow-up by chest and abdomen CT after 3, 6, 12, 18, 24, 36, 48, and 52 months. No recurrence was noted in four patients, although one patient had local lymph node recurrence at station 9, 11, and 12 and therefore underwent chemotherapy. PET-CT FDG was positive in only one patient (SUV max 2.3) for the lung nodule.

### Identification of Tumor Endothelial Cells With High Aldehyde Dehydrogenase Activity

**Figure 1:**
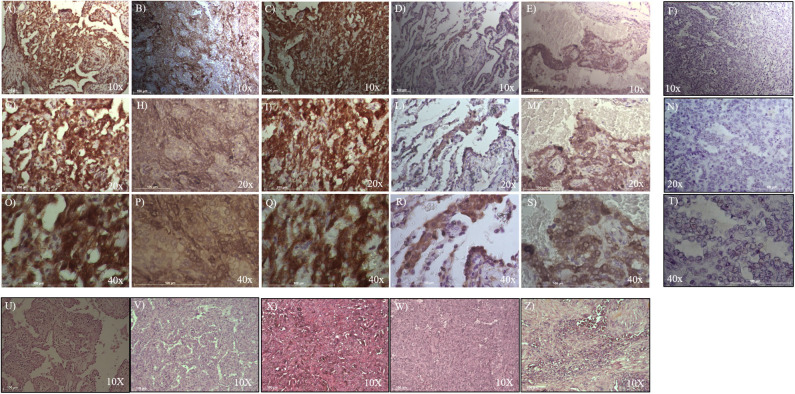
Immunohistochemical analysis of ALDH positive cells in five cases of pneumocytoma of the lung. Tissue sections were stained with anti-ALDH antibody to detect the positivity in these five different cases of pneumocytoma of the lung. **(A–O)** Case 1, **(B–P)** Case 2, **(C–Q)** Case 3, **(D–R)** Case 4, **(E–S)** Case 5. Semi-quantitative method was used to assess the ALDH positivity of the tumor cells: 0 (<5% positive), 1 (5–25% positive), 2 (>25–50% positive), 3 (>50–75% positive), and 4 (>75% positive). Representative images of the five cases, stained for ALDH, are shown at 10, 20, and 40x magnification. Representative images corresponding to each tumor samples stained with hematoxylin and eosin **(U, V, X, W, Z)** are shown at 10x magnification. Isotype control for ALDH antibody was shown **(F, N, T)**. Scale bar = 100 μm.

**Figure 2:**
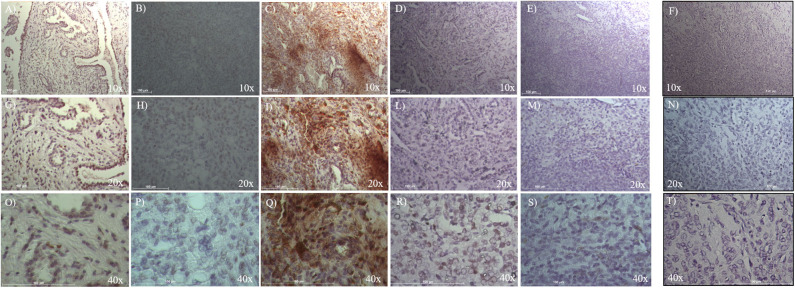
Immunohistochemical analysis of SOX-2 positive cells in five cases of pneumocytoma of the lung. Tissue sections were stained with anti-SOX-2 antibody to detect the positivity in these five different cases of pneumocytoma of the lung. **(A–O)** Case 1, **(B–P)** Case 2, **(C–Q)** Case 3, **(D–R)** Case 4, **(E–S)** Case 5. Semi-quantitative method was used to assess the SOX-2 positivity of the tumor cells: 0 (<5% positive), 1 (5–25% positive), 2 (>25–50% positive), 3 (>50–75% positive), and 4 (>75% positive). Representative images of the five cases are shown at 10, 20, and 40x magnification. Isotype control for SOX-2 antibody was shown **(F, N, T)**. Scale bar = 100 μm.

To evaluate ALDH and SOX-2 positivity in these five cases of pneumocytoma, immunohistochemistry was performed and a score value was then assigned, as previously described (25). Tissue sections were examined at 10, 20, and 40x magnification to assign the appropriate values. ALDH-positive cells from tissue slides, represented by brown cytoplasm, and SOX-2 positive cells by brown nuclei, were both analyzed at 10, 20, and 40x magnification (Figures 1, 2). Three of the five cases of pneumocytoma showed an ALDH score value of 4, while the other two cases had a score value of 3. On the other hand, for three of the five cases of pneumocytoma, SOX-2 score value was 0, despite one case having a score value of 3 while the last case had a score value of 1.

To analyze whether ALDH is expressed in human pneumocytoma, an immunohystochemical staining was performed on the paraffin-embedded sections of the tumor patient. Tissue sections were examined at 10, 20, and 40x magnification to assign the appropriate values. ALDH staining was strongly positive in three of the five patient cases, which had a score value of 4 (Figures 1B–R, Table 1). Regarding the other two patients, ALDH was strongly positive, with a score value of 3 (Figures 1A–O,S, Table 1).

**Table 1:** Patients characteristics.

**Patient**	**Tumor hystotype and characteristic**	**ALDH expression**	**SOX-2 expression**
Pt#1	Pneumocytoma	3	0
Pt#2	Pneumocytoma	4	1
Pt#3	Pneumocytoma	4	3
Pt#4	Pneumocytoma	4	0
Pt#5	Pneumocytoma with lymphonodes local recurrence	3	0

*This table represents the five patients case of pneumocytoma analyzed for the immunohystochemical expression of ALDH and SOX-2. A score value for ALDH and SOX-2 was assigned using the following score classes: 0 (<5% positive), 1 (5–25% positive), 2 (>25–50% positive), 3 (>50–75% positive), and 4 (>75% positive)*.

### Identification of Cells Expressing Stem Cell Factor SOX-2

To evaluate SOX-2 positivity in these five cases of pneumocytoma, immunohistochemistry was performed, and a score value was assigned as previously described (25). Tissue sections were examined at 10, 20, and 40x magnification to assign the appropriate values. SOX-2 positive cells were represented by brown nuclei and were analyzed at 10, 20, and 40x magnification (Figure 2). Three of the five patient cases of pneumocytoma had a SOX-2 score value equal to 0 (Figures 2A-S, Table 1), while the remaining two patients had a score value of 3 (Figures 2C–Q) and a score value of 1 (Figures 2B–P, Table 1).

## Discussion

Pulmonary sclerosing pneumocytoma (PSP) is a rare benign tumor typically occurring in women with a favorable prognosis. Recurrence for this disease is infrequent, although not impossible to find. The nomenclature for this benign disease (8–10) has recently been renamed “pulmonary sclerosing pneumocytoma” (6).

It is extremely difficult to obtain an accurate diagnosis for PSP due to its low incidence and the impossibility of establishing a clinical and radiological differential diagnosis from other diseases. This is well-documented in the scientific literature where surgery is the gold standard treatment and the most common immunohistochemistry markers are used for diagnosis (Table 2) (15). Moreover, due to the frequent expression in females, estrogen receptors have been studied using immunohistochemistry, which has revealed an overexpression of ERbeta in 91.9% of patients affected by the disease (19, 20). The frequent markers used for diagnosis indicate positivity for EMA (epithelial membrane antigen), TTF-1 (transcription termination factor 1), and CK-7 (cytokeratin-7) staining (Table 2) (18, 26–35). However, currently no specific markers in this type of benign tumor have been identified.

**Table 2:** Review of the literature regarding PSP of the lung.

**Author/year**	**Patients enrolled (n)**	**Gender (M/F)**	**Age (Mean)**	**Type of treatment**	**IHC marker**	**Methastatic lymphonodes**	**Recurrence**
Hu et al. (26)	46	5 M 41 F	51.4	45/46 Surgery −21 enucleation −9 wedge resections −15 lobectomies	/	/	No
Devouassoux- Shisheboran et al. (18)	100	17 M 83 F	46	54/100 Surgery: 5 wedge resections 37 lobectomies 2 bilobectomies 1 pneumonectomy - 9 procedures not specified specificata	-pancytokeratin+; CAM 5.2+, CK7 +, EMA + calretinin –; CK5/6 – TTF1 +; Sp A e B + chromogranin –; synaptophysin –; Leu 7 – - S100 -	Yes, 1 case	No
Shin et al. (27)	76	9 M 67 F	50	45/76 Surgery: 33 segmentectomies 11 lobectomies - 1 bilobectomies	TTF1+, cytokeratin +, EMA+; - ki-67–	/	/
Yang et al. (28)	59	2 M 57 F	49.85	Wedge resection (58 wedge resections)	Surface cell AE1/AE3+ e CK7+; Stromal cell AE1/AE3- e CK7+; - TTF1+ e EMA+	No	No
Lei et al. (29)	28	3 M 25 F	46.1	13 enucleation 7 wedge resection 8 lobectomies	/	No	Yes
Park et al. (30)	32	1 M 31 F	47.8	19 lobectomies 5 wedge resections 1 segmentectomy 7 wedge resections	/	Yes, 1 case	No
Cho et al. (31)	11	1 M 10 F	48.6	Surgery	MMP-9 + Tubulinα + IGF1 +	/	/
Schmidt (32)	6	/	/	/	TTF1 + Napsin A +	/	/
Wu et al. (33)	18	2 M 16 F	44.6	/	P40 + TTF1+, SHL+, EMA+, Vimentin+	No	No
Lovrenski et al. (34)	6	1 M 5 F	/	Surgery	TTF1+ e panCK +	No	No

*This Table shows treatments and immunohistochemical characteristics. ALDH and SOX-2 have been never considered*.

Our study is the first attempt to show the presence of ALDH and SOX-2 in pulmonary sclerosing pneumocytoma of the lung. In particular, we decided to test ALDH and SOX2 in our PSP samples for the well-known vascular aspect of this tumor which may be associated with the role that these two markers have into the tumor vascularization process. Recently, it has been revealed that tumor endothelial cells were different from normal endothelial cells in various aspects such as gene expression profiles and for the activities of the ALDH, enzyme that plays a key role in the metabolism of aldehydes. Several studies showed that different type of stem cell types including hematopoietic stem cells and neural stem cells possess high ALDH activities. Thus, ALDH is used extensively as a stem cell marker, also for endothelial stem-like cells (22). Scientific advantages have shown that ALDH is upregulated in human endothelial cells *in vivo* and may be involved in tumor angiogenesis in cancer patients (36). There is likely an important role for ALDH in the formation of this tumor, which derives from vascular endothelial cells. On this marked trail, our results show a very high expression of ALDH (score of 4) in three patients and a high expression (score of 3) in the other two patients. Additionally, we found that the positivity of ALDH was sparsely distributed within the tumor, which may support endothelial cell heterogeneity. This concept may be attributed to the fact that ALDH expression levels are linked to the tumor microenvironment or tumor malignancy, which may affect ALDH expression in the endothelial cells themselves (37). Furthermore, the evaluation of SOX-2 in our patients' tumors derived from the knowledge that SOX-2 is known to play an important role in vascular development and disease, since its transcription factors have been shown to be essential mediators in vascular development, and in the developing endothelium (23). The involvement of SOX-2 may be crucial also for the angiogenesis in this type of tumor, synergistically participating with ALDH positive cells in the vascular impairment of pneumocytoma (22). Interestingly, SOX-2 was found to be highly expressed (score of 3) in only one patient: the same patient showing an ALDH score of 4 and lymph node recurrence, which is highly uncommon for this disease (24).

Although we are conscious of the fact that our study focuses on a small number of patients, due to the rarity of this benign disease of the lung, we strongly believe that the presence of ALDH and SOX-2 in pneumocytoma of the lung requires deeper analysis, especially in patients who show recurrence. This will be helpful for understanding the molecular mechanisms behind the phenomenon, and for developing future targeted therapies, which may be more effective than surgery in removing or controlling the disease.

## Data Availability Statement

The raw data supporting the conclusions of this article will be made available by the authors, without undue reservation.

## Ethics Statement

This Study has been approved by the Ethics Committee at University Hospital of Modena, MODENA, Italy, on 4 June 2019, Prot. N. 395/2019/OSS/AOUMO. The patients/participants provided their consent to participate in this study.

## Author Contributions

The idea for the manuscript was conceived in February 2019 by BA and was further developed by VM, DB, BM, FBa, RD'A, FBe, MD, UM, and AM was involved in histopathological diagnosis. BA and VM wrote the first draft of the manuscript. BA and UM have been involved in surgery and tissue collection. VM performed laboratory experiments. BA, VM, FBa, MD, FBe, RD'A, AM, and UM all reviewed the manuscript and were involved in its critical revision before submission. All authors read and approved the final manuscript.

## Conflict of Interest

The authors declare that the research was conducted in the absence of any commercial or financial relationships that could be construed as a potential conflict of interest.

## References

[1] Yalcin B Bekci TT Kozacioglu S Bolukbas O. Pulmonary sclerosing pneumocytoma, a rare tumor of the lung. Respir Med Case Rep. (2019) 26:285–7. 10.1016/j.rmcr.2019.02.002 30847275 PMC6389774

[2] Liebow AA Hubbell DS. Sclerosing hemangioma (histiocytoma, xanthoma) of the lung. Cancer. (1956) 9:53e75. 13284701 10.1002/1097-0142(195601/02)9:1<53::aid-cncr2820090104>3.0.co;2-u

[3] Hishida T Yoshida J Nishimura M Ishii G Nishiwaki Y Nagai K. Multiple sclerosing hemangiomas with a 10-year history. Jpn J Clin Oncol. (2005) 35:37e9. 10.1002/1097-0142(195601/02)9:1<53::AID-CNCR2820090104<3.0.CO;2-U 15681603

[4] Travis WD Brambilla E Nicholson AG Yatabe Y Austin JHM Beasley MB. The 2015 World Health Organization of lung tumors. J Thorac Oncol. (2015) 10:1243–60. 10.1097/JTO.0000000000000630 26291008

[5] Cheung YC Ng SH Chang JWC Tan CF Huang SF Yu CT. Histopathological and CT features of pulmonary sclerosing haemangiomas. Clin Radiol. (2003) 58:630e5. 10.1016/S0009-9260(03)00177-6 12887957

[6] Cardemil G Fernández E Riffo P Reyes D Ledezma R Mira M. Sclerosing hemangioma presenting as a solitary lung nodule. report of one case. Rev Med Chile. (2004) 132:853–6. 10.4067/S0034-98872004000700010 15379333

[7] Keylock CPTJB Galvin JR Franks TJ. Sclerosing hemangioma of the lung. Arch Pathol Lab Med. (2009) 133:820–5. 10.1043/1543-2165-133.5.820 19415961

[8] Danciu M Lunguleac T Grigorescu C. Incidental finding of a sclerosing hemangioma in a Caucasian woman. Rom J Morphol Embryol. (2015) 56:545–8. 26193226

[9] Haimoto H Tsutsumi Y Nagura H Nakashima N Watanabe K. Immunohistochemical study of so-called sclerosing haemangioma of the lung. Vichows Arch A Pathol Anat. (1985) 407:419–30. 10.1007/BF00709988 2413615

[10] Nakanishi K Kohzaki S Fujimoto S Horita Y Hayashi K. Pulmonary sclerosing hemangioma: report of a case with emphasis on dynamic MR imaging findings. Radiat Med. (1997) 15:117–9. 9192438

[11] Iyoda A Hiroshima K Shiba M Haga Y Moriya Y Sekine Y. Clinicopathological analysis of pulmonary sclerosing hemangioma. Ann Thorac Surg. (2004) 78:1928–31. 10.1016/j.athoracsur.2004.05.069 15561002

[12] Sugio K Yokoyama H Kaneko S Ishida T Sugimachi K. Sclerosing hemangioma of the lung: radiographic and pathological study. Ann Thorac Surg. (1992) 53:295–300. 10.1016/0003-4975(92)91336-8 1309991

[13] Chung MJ Lee KS Han J Sung YM Chong S Kwon OJ. Pulmonary sclerosing hemangioma presenting as solitary pulmonary nodule: dynamic CT findings and histopathologic comparisons. Am J Roentgenol. (2006) 187:430–7. 10.2214/AJR.05.0460 16861548

[14] Im JG Kim WH Han MC Han YM Chung JW Ahn JM. Sclerosing hemangiomas of the lung and interlobar fissures: CT findings. J Comp Assist Tomogr. (1994) 18:34–8. 10.1097/00004728-199401000-00007 8282879

[15] Xie RM Zhou XH Lu PX He W. Diagnosis of pulmonary sclerosing hemangioma with incremental dynamic CT: analysis of 20 cases. Chin J Tuberculosis Respir Dis. (2003) 26:7–9. 12775260

[16] Lin H Yao H Peng F CT image morphology features of pulmonary sclerosing hemangiomas. Chin Ger J Clin Oncol. (2011) 10:19–23. 10.1007/s10330-011-0727-5

[17] Wang QB Chen YQ Shen JJ Zhang C Song B Zhu XJ. Sixteen cases of pulmonary sclerosing haemangioma: CT findings are not definitive for preoperative diagnosis. Clin Radiol. (2011) 66:708–14. 10.1016/j.crad.2011.03.002 21529795

[18] Devouassoux-Shisheboran M Hayashi T Linnoila RI Koss MN Travis WD. A clinicopathologic study of 100 cases of pulmonary sclerosing hemangioma with immunohistochemical studies: TTF-1 is expressed in both round and surface cells, suggesting an origin from primitive respiratory epithelium. Am J Surg Pathol. (2000) 24:906–16. 10.1097/00000478-200007000-00002 10895813

[19] Liu W Tian XY Li Y Zhao Y Li B Li Z. Coexistence of pulmonary sclerosing hemangioma and primary adenocarcinoma in the same nodule of lung. Diagn Pathol. (2011) 6:41–6. 10.1186/1746-1596-6-41 21599956 PMC3117760

[20] Hara M Iida A Tohyama J Miura N Shiraki N Itoh M. FDG-PET findings in sclerosing hemangioma of the lung: a case report. Rad Med. (2001) 19:215–8. Available online at: https://europepmc.org/article/med/11550723 11550723

[21] Timpone V Danielson D Woods A Clark B FDG–PET imaging findings of a pulmonary sclerosing hemangioma. Eur J Radiol Extra. (2011) 79:e65–7. 10.1016/j.ejrex.2011.05.003

[22] Ohmura-Kakutani H Akiyama K Maishi N Ohga N Hida Y Kawamoto T. Identification of tumor endothelial cells with high aldehyde dehydrogenase activity and a highly angiogenic phenotype. PLoS ONE. (2014) 9:e113910. 10.1371/journal.pone.0113910 25437864 PMC4250080

[23] Yao Y Yao J Boström KI Martin KA. SOX Transcription Factors in Endothelial Differentiation and Transitions. Front Cardiovasc Med. (2019) 6:30. 10.3389/fcvm.2019.00030 30984768 PMC6447608

[24] Horiguchi K Fujiwara K Yoshida S Tsukada T Hasegawa R Takigami S. CX3CL1/CX3CR1-signalling in the CD9/S100β/SOX2-positive adult pituitary stem/progenitor cells modulates differentiation into endothelial cells. Histochem Cell Biol. (2020) 153:385–96. 10.1007/s00418-020-01862-0 32152663

[25] Kahlert C Bergmann F Beck J Welsch T Mogler C Herpel E. Low expression of aldehyde dehydrogenase 1A1 (ALDH1A1) is a prognostic marker for poor survival in pancreatic cancer. BMC Cancer. (2011) 11:275. 10.1186/1471-2407-11-275 21708005 PMC3135572

[26] Hu AM Zhao D Zheng H Wang QH Lyu Y Li BL. Preoperative diagnosis in 46 cases of pulmonary sclerosing hemangioma. Chin Med J. (2016) 129:1377–8. 10.4103/0366-6999.182839 27231179 PMC4894052

[27] Shin SY Kim MY Oh SY Lee HJ. Pulmonary sclerosing pneumocytoma of the lung : CT characteristics in a large series of a tertiary referral center. Medicine. (2015) 94:e498. 10.1097/MD.0000000000000498 25634202 PMC4602969

[28] Yang C Lee L. Pulmonary sclerosing pneumocytoma remains a diagnostic challenge using frozen sections : a clinicopathological analysis of 59 cases. Histopathology. (2018) 72:500–8. 10.1111/his.13391 28881050

[29] Lei Y Yong D Jun-zhong R Zhi Y Zi-tong W. Treatment of 28 patients with sclerosing hemangioma (SH) of the lung. J Cardiothorac Surg. (2012)7:34. 10.1186/1749-8090-7-34 22515818 PMC3377544

[30] Park JS Kim K Shin S Shim H Kim HK. Surgery for pulmonary sclerosing hemangioma : lobectomy versus limited resection. Korean J Thorac Cardiovasc Surg. (2011)44:39–43. 10.5090/kjtcs.2011.44.1.39 22263122 PMC3249271

[31] Cho SJ Lian JJ Kim B-Y Cho S-J Jung WY Han J-H. Increased expression of matrix metalloproteinase 9 and tubulin- α in pulmonary sclerosing hemangioma. Oncol Rep. (2007) 18:1139–44. 10.3892/or.18.5.1139 17914564

[32] Schmidt LA Myers JL Mchugh JB. Napsin a is differentially expressed in sclerosing hemangiomas of the lung. Arch Pathol Lab Med. (2012) 136:1580–4. 10.5858/arpa.2011-0486-OA 23194051

[33] Wu J Zhang C Qiao H. The significance of p40 expression in in sclerosing hemangioma of lung. Sci Rep. (2014) 4:6102. 10.1038/srep06102 25130377 PMC4135335

[34] Lovrenski A Vasilijević M Panjković M Tegeltija D Vučković D Baroš I. Sclerosing pneumocytoma: a ten-year experience at a western balkan university hospital. Medicina. (2019) 55:27. 10.3390/medicina55020027 30691016 PMC6409643

[35] Jungraithmayr W Eggeling S Ludwig C Kayser G Passlick B. Sclerosing hemangioma of the lung: a benign tumour with potential for malignancy? Ann Thorac Cardiovasc Surg. (2006) 12:352–4. Available online at: http://www.atcs.jp/pdf/2006_12_5/352.pdf 17095978

[36] Corti S Locatelli F Papadimitriou D Donadoni C Salani S Del Bo R. Identification of a primitive brain-derived neural stem cell population based on aldehyde dehydrogenase activity. Stem Cells. (2006) 24:975–85. 10.1634/stemcells.2005-0217 16293577

[37] Hida K Maishi N Akiyama K Ohmura-kakutani H Torii C Ohga N. Dehydrogenase activity show drug resistance. Cancer Sci. (2017) 108:2195–203. 10.1111/cas.13388 28851003 PMC5666026

